# Panton-Valentine Leukocidin-Producing *Staphylococcus aureus* Infection: A Case Series

**DOI:** 10.3390/idr12030014

**Published:** 2020-11-03

**Authors:** Beatriz Prista-Leão, Isabel Abreu, Raquel Duro, André Silva-Pinto, Filipa Ceia, Paulo Andrade, Joana Sobrinho-Simões, Margarida Tavares, José Manuel Pereira, Lurdes Santos, António Sarmento

**Affiliations:** 1 Department of Infectious Diseases, Centro Hospitalar Universitário de São João, Alameda Prof. Hernâni Monteiro, 4200-319 Porto, Portugal; isabelgomesabreu@gmail.com (I.A.); raquel.duro@gmail.com (R.D.); pintoandre@gmail.com (A.S.-P.); fsfceia@gmail.com (F.C.); andrade.pf@gmail.com (P.A.); maria.lurdes.uci@gmail.com (L.S.); antonio.sarmento@hsjoao.min-saude.pt (A.S.); 2 Faculty of Medicine, University of Porto, Alameda Prof. Hernâni Monteiro, 4200-319 Porto, Portugal; 3 Prevention and Control of Infection and Antimicrobial Resistance Unit, Centro Hospitalar Universitário de São João, 4200-319 Porto, Portugal; 4 Molecular Biology Laboratory of the Department of Clinical Pathology, Centro Hospitalar Universitário de São João, 4200-319 Porto, Portugal; jssimoes@hsjoao.min-saude.pt; 5 Department of Pediatrics, Centro Hospitalar Universitário de São João, 4200-319 Porto, Portugal; margaridamtavares47@gmail.com; 6 Department of Intensive Medicine, Centro Hospitalar Universitário de São João, 4200-319 Porto, Portugal; jmcrpereira@yahoo.com

**Keywords:** Panton-Valentine leukocidin, *Staphylococcus aureus*, toxin, virulence factor

## Abstract

Panton-Valentine leukocidin-producing *Staphylococcus aureus* (PVL-SA) is associated with relapsing multifocal skin and soft tissue infections (SSTI), necrotizing pneumonia (NP) and severe musculoskeletal infections. Epidemiology is underknown and underdiagnosis is likely. Recent travel abroad, case clustering and relapsing disease are often reported. We reviewed all cases of PVL-SA infection diagnosed at our center, and found 21 cases over a 43-month period. Most patients were adult males, had relevant travel history, reported recurrent disease and presented with SSTI. Etiologic diagnosis took up to five years; meanwhile, 42% of patients had antibiotic treatments. Draining procedures were required in 43% of patients and intensive care support in 19%. All patients recovered. Methicillin-resistance prevalence was 24%. Only 2/13 decolonized patients had posterior relapsing SSTI, both with likely infected contacts. PVL-SA infection’s severity and impact are clear, even in small case series as ours. Physician awareness and active PVL-gene search are crucial for an adequate management.

## 1. Introduction

Panton-Valentine leukocidin-producing *Staphylococcus aureus* (PVL-SA) is a recognized cause of relapsing multifocal skin and soft tissue infections (SSTI), necrotizing pneumonia (NP) and severe musculoskeletal infections, particularly in children [1,2]. Relapse after an adequate treatment is common in the absence of decolonization [2,3]. Suspicion may arise from some characteristic features (e.g., severity/extent of disease, recurrence, clustering), but confirmed diagnosis requires specific non-routine tests. Considering its growing prevalence [4,5,6], understanding PVL-SA’s particular features and risk factors is required for a better clinical practice.

In this work, we characterize all recorded cases of PVL-SA infection diagnosed in a Portuguese tertiary care center.

We analyzed the Molecular Biology Laboratory database and selected every patient with positive samples for PVL-SA since 27 April 2016, when the first PVL-gene detection test performed, until 30 November 2019. In our hospital, PVL-gene investigation is carried out by in-house real-time polymerase chain reaction on pure cultures of *S. aureus* [7], upon specific medical request, in the event of clinical suspicion.

The institutional electronic database was revised, and demographic, clinical and microbiological data were retrospectively collected.

The study was conducted in accordance with the Declaration of Helsinki and approved by the local Ethics Committee.

## 2. Case Reports

Twenty-one patients were identified: 14 were male, and ages ranged between 14 and 65 years old (median 41 ± 14). The main demographic, clinical and microbiological characteristics are presented in Table 1.

Nine patients had medical comorbidities, seven with immunological impairment: type 2 diabetes mellitus (DM) (3/21), human immunodeficiency virus (HIV) infection (2/21) and autoimmune disease (2/21).

Most patients (17/21) presented with SSTI: 14/17 had multiple or recurrent furunculosis and/or cutaneous abscess and 3/17 presented with cellulitis or phlegmon; 7/17 required a draining intervention; 4/17 were admitted for intravenous antibiotics.

Severe disease requiring Intensive Care Unit (ICU) admission occurred in a child with osteomyelitis and compartment syndrome of the lower limb and in three adult patients with NP. The child required emergent debridement; NP patients required empyema drainage (1/3), venovenous extracorporeal membrane oxygenation (VV-ECMO) (2/3) and renal replacement therapy (RRT) (1/3). All of them fully recovered.

Relative neutropenia was detected in two patients, both with mild SSTI.

From the first symptoms to the identification of PVL-SA, it took between three days and five years (median 18 ± 407 days); all severe cases took less than a week.

Recurrent disease was restricted to SSTI patients: 7/17 had up to four episodes of active disease and 4/17 had five or more. Nine patients had already been treated with systemic antibiotics in previous episodes.

Two secondary cases were documented, both studied after interviewing the index case, who reported a suggestive clinical picture (recurrent furunculosis) in her parents. Five other probable secondary cases were reported in clinical records (all with recurrent furunculosis), but sampling for microbiological study before successful decolonization was not possible.

Recent travel before onset of symptoms was reported by 12/21 patients. 

A healthcare associated context was reported in 2/21 patients.

Most isolates (16/21) were methicillin-sensitive *Staphylococcus aureus* (MSSA). Methicillin-resistance was relatively more frequent in patients with previous antibiotic treatment (3/9 vs. 2/12, *p* = 0.03). Resistance to sulfamethoxazole/trimethoprim (6/21) and clindamycin (3/21) and other details are presented in Table 2 and Table 3.

A β-lactam antibiotic, mostly flucloxacillin, was used in 14/21 patients. Combination treatment was used in 3/21 cases (clindamycin plus flucloxacillin, ceftriaxone or linezolid). De-escalation of antimicrobial therapy was performed in one patient; all other patients completed successful treatment with the empirically started antibiotic. Treatment data were not available for one patient.

After clinical resolution, 13/21 patients performed decolonization. Decolonization was not prescribed in 4/21 patients, and 4/21 were lost to follow up. Following decolonization, 2/13 patients had recurrence of SSTI. Both patients reported having a close relative with history of multiple furunculosis who did not follow decolonization recommendations. After repeating decolonization simultaneously with their household, none experienced recurrence.

## 3. Discussion

According to other series, PVL-SA presented mostly as SSTI in young immunocompetent adults [1,4,5,8,9]. Male gender was predominant, in contrast with what was described previous works [4].

Comorbidities such as atopy or smoking may increase risk of PVL-SA infection. [4] Conditions affecting immune function were prevalent in our patients, but some conditions or habits may have been underreported in clinical records.

PVL-SA infections have three major clinical manifestations: SSTIs, NP and severe musculoskeletal infection [1,2,8].

Compared with PVL-negative *S. aureus* SSTIs, PVL-SA SSTIs have some distinctive features: lesions often become rapidly extensive with pronounced inflammation; suppurative necrosis and deeper tissue involvement (such as furuncles, carbuncles and skin abscesses); frequent need for invasive draining procedures; and high risk of transmission between close contacts [2,3,4,8,9,10,11]. PVL-SA’s hallmark clinical syndrome is recurrent SSTI, which is reported in up to 90% of patients, often over a significant period of time [2,3,8,11]. Indeed, SSTI was the most common presentation in our patients, and recurrent disease occurred in 65% of them.

NP, on the other hand, is one of the most severe presentations of PVL-SA infection. Typically, a flulike syndrome prodrome is followed by rapid onset of fever, respiratory deterioration and sepsis, with progression to acute respiratory distress syndrome, often requiring ventilatory support, and occasionally with hemoptysis, progressive multilobar consolidation, pleural effusion and cavitary infiltrates. [2,5,8] Neutropenia has also been reported in these patients [1]. NP has been reported to be more common in children and young otherwise healthy adults [2,7,12]. Our NP patients were middle-aged males with significant comorbidities; none of them had neutropenia. In accordance with the expected severity of disease, all were admitted to an ICU with a significant need for organ support. Even though the reported mortality is up to 75% [5,8,12], all three patients fully recovered.

PVL-SA is also associated with severe musculoskeletal infections, mostly in children [1,2,8], presenting with aggressive local disease (e.g., large periosteal or multifocal abscesses, pyomyositis, and deep vein thrombosis) and intense systemic inflammation [1,8,13]. Patients typically present acutely unwell, with severe sepsis, often with bacteremia and metastatic infection, requiring surgery, ICU admission and prolonged hospital stay, along with a high risk of long-term sequelae [1,2,8,13,14]. The only pediatric case in our series presented with septic shock secondary to rapidly progressive cellulitis, osteomyelitis and compartment syndrome of the lower limb. He was hospitalized for 54 days, but recovered without sequelae.

PVL-SA disease may extend over weeks to months, with a history of multiple and recurrent skin lesions, often with evident clustering [1,2,3,5,11]. In our study, disease persisted for as long as 5 years, and 43% of patients had systemic antibiotic trials, with no or only temporary effect. Until identification of the agent and adequate secondary prevention measures take place, great morbidity may be associated with these infections. Hence, PVL-SA SSTI should be considered in all patients with a history of recurrent or indwelling SSTIs, particularly when distinct close contacts are affected, or when recurrence follows a spontaneous or antibiotic-induced remission. Moreover, previous skin lesions are reported by 25% of patients with PVL-SA pneumonia, either in the patients themselves or in close contacts [1,5], which reinforces a possible role for early diagnosis in the prevention of persistent and also severe disease. History of skin lesions was not reported nor excluded in clinical records of our severe patients.

Although the lack of epidemiological surveillance results in inaccurate estimates, PVL-SA is probably rare in Europe (prevalence ≤10%) and endemic in Africa (prevalence ≤74%) [2,3,6,8,10,15,16,17]. However, reported PVL-SA infections have been increasing recently, both in Europe and USA [4,5,6,7,17,18]. Greater awareness and effort towards a correct diagnosis are certainly relevant, but intercontinental travelers may also contribute. In fact, travelers have higher reported prevalence of SSTIs when compared to the general population, mostly imported from Africa and South America, possibly by exposure to *S. aureus* strains to which they have low immunity [3,10,11]. Longer stay, humanitarian work and visiting friends/relatives may pose higher risk for PVL-SA acquisition [3]. Accordingly, in our study, 12/21 patients had relevant travel history, mostly to Africa, and only 2/12 stayed for <2 weeks. In an era of growing population mobility, the prevalence of imported infections may keep rising and, therefore, travel history must be investigated and may be a valuable clue to PVL-SA infection suspicion.

PVL-SA induces severe inflammatory response, tissue damage and necrosis, with higher propensity for abscess formation and risk of poorer antibiotic diffusion, which may result in local subinhibitory concentrations [2,8,19]. Purulent collection drainage is imperative and reduces both bacterial and toxin load [2,3,8]. Invasive focus control was required in 9/21 patients, namely through thoracic empyema drainage and emergency debridement surgery.

Antibiotic use is consensual in PVL-SA infections management, but the best regimen is still undefined [1,2,8]. While above the minimal inhibitory concentration (MIC), β-lactam antibiotics act both by killing bacteria and inhibiting PVL production; below the MIC, they may actually enhance PVL production [8,17], a risk that may be overcome by adding clindamycin or linezolid to the treatment [8,20]. However, even though PVL positivity appears to occur independently of antibiotic resistance, resistance to non-β-lactam antibiotics seems to be common in imported *S. aureus* [3,4,18].

MRSA was found in 5/21 patients, and resistance to other SSTI first-line treatments, such as trimethoprim/sulfamethoxazole or clindamycin, was documented in 6/21 and 3/21, respectively. Considering the fact that we found no outcome differences between antibiotic regimens, we support that local epidemiology and guidelines should be taken into account and that, in the absence of a high MRSA prevalence, standard therapy with adequate doses of anti-staphylococcal β-lactams is a valuable choice [2]. Association between PVL-SA infection and the need for hospitalization is controversial, and predictors of poor outcome remain largely unknown [3,4,8,17]. In our study, a third of patients actually required inpatient treatment, including 4/7 who were admitted to ICU. All of them fully recovered, without sequelae. PVL positivity may indeed be related to more aggressive disease, but we believe that the decision on the best place for treatment depends more on the clinical severity or need for invasive intervention, rather than on PVL positivity/negativity.

Case clustering (in household, sexual partners, social or professional activities, sports, prisons, etc.) is a PVL-SA hallmark [1,3,4,5,8,9,11]. We included in the study two documented cases of secondary PVL-SA infection, and, from clinical records, five other plausible secondary cases of infection were found. Other cases may have been under-investigated or underreported in clinical records.

PVL-SA carriage (typically in the oropharynx, axilla or perineum, rather than nasal mucosa) is an established risk factor for active disease, infection persistence, recurrent SSTI and also for its dissemination [2,3,5,8,10,11]. Thus, although clear evidence of the impact of decolonization in the prevention of PVL-SA infection is lacking, topical decolonization therapy (e.g., chlorhexidine bath and mouthwash combined with nasal mupirocin for five days) may be beneficial, and extension to close contacts (either empirically or after positive screening) should be considered [1,2,3,5,8,10]. Decolonization should take place after treatment and resolution of all active lesions and should be performed simultaneously in all household members [1,2]. Failure of eradication or recolonization and recurrent disease are not uncommon, and could be explained by non-compliance with recommendations, presence of infected devices or by colonized close contacts or fomites, among other factors [2]. Accordingly, only 2/13 decolonized patients had posterior recurrence, and both of them admitted having close relatives with history of multiple furunculosis who did not follow recommendations. After correct and simultaneous decolonization, disease did not recur. Identification of relevant close contacts is, therefore, of uttermost importance and may prevent both important morbidity and possible serious future episodes of disease.

Our study has several limitations: due to its retrospective nature, relevant data, such as information about predisposing risk factors, therapeutic issues and secondary cases may be lacking. Additionally, as the PVL gene is not routinely investigated, it is possible that milder PVL-SA infection cases are underrepresented and that we have only had access to patients whose presentation was sufficiently severe, exuberant or recurrent, to make the clinician suspect a PVL-SA infection and request this test.

## 4. Conclusions

The severity and impact of PVL-SA infections are clear, even in small case series such as ours: 19% of patients had severe and potentially fatal infection, and 52% had recurrent disease before definitive diagnosis.

Regional incidence of PVL-SA infections is unknown, and probably underdiagnosed. Therefore, it is crucial to have a high suspicion index in the presence of recurrent and/or clustered SSTIs, particularly if risk factors, such as travel history, are present.

We believe that, after treatment, decolonization may be beneficial to patients and close contacts and efforts are being made in our center to standardize these.

## Figures and Tables

**Table 1:** Characterization of patients with Panton-Valentine leukocidin-producing *Staphylococcus aureus* (PVL-SA) infection.

		Number of Patients (%)		
Characteristics	All Patients	Mild Disease: Multiple/Recurrent Furunculosis Or Skin Abscesses	Moderate Disease: Cellulitis or Phlegmon	Severe Disease: Septic Shock + Necrotizing Pneumonia or Osteomyelitis
All patients	21/21 (100)	14/21 (67)	3/21 (14)	4 */21 (19)
Male gender	14/21 (67)	9/14 (64)	2/3 (67)	3/4 (75)
Relevant comorbidities				
Diabetes mellitus	3/21 (14)	2/14 (14)	0	1/4 (25)
HIV	2 ^°^/21 (10)	1/14 (7)	1/3 (33)	0
Autoimmune disease	2 ^#^/21 (10)	1/14 (7)	0	1/4 (25)
Asthma	1/21 (5)	0	1/3 (33)	0
Intravenous drug use	1/21 (5)	0	0	1/4 (25)
Type of case				
Primary	19/21 (90)	12/14 (86)	3/3 (100)	4/4 (100)
Secondary	2/21 (10)	2/14 (14)	0	0
Recent travel history				
None	9/21 (63)	2/14 (14)	2/3 (67)	4/4 (100)
European countries, other than Portugal	1 ^§^/21 (5)	1/14 (7)	0	0
Africa	8 ^^^/21 (38)	8/14 (57)	0	0
Asia	2 ^$^/21 (10)	1/14 (7)	1/3 (33)	0
USA	1/21 (5)	1/14 (7)	0	0
Purpose of travel, if any				
Birthplace and country of residence	4/12 (33)	4/11 (36)	0	0
Visiting friends and family	3/12 (25)	3/11 (27)	0	0
Business	2/12 (17)	2/11 (18)	0	0
Leisure	2/12 (17)	1/11 (9)	1/1 (100)	0
Humanitarian work	1 (8)	1/11 (9)	0	0
Length of the disease until microbiological diagnosis				
Number of episodes				
1	10/21 (48)	4/14 (29)	2/3 (67)	4/4 (100)
2 to 4	7/21 (33)	7/14 (50)	0	0
5 or more	4/21 (19)	3/14 (21)	1/3 (33)	0
Number of systemic antibiotic treatments				
1	12 (57)	6/14 (43)	2/3 (67)	4/4 (100)
2 to 4	5 (24)	5/14 (36)	0	0
5 or more	4/21 (19)	3/14 (21)	1/3 (33)	0
Relative neutropenia **	2/21 (9,5)	2/14 (14)	0	0
Biological sample from which PVL-SA was recovered				
Pus	18 ^°°^/21 (86)	14/14 (100)	3/3 (100)	1/4 (25)
Respiratory samples	2/21 (10)	0	0	2/4 (50)
Pleural fluid	1/21 (5)	0	0	1/4 (25)
Need for invasive procedures	9/21 (43)	4/14 (29)	3/3 (100)	2/4 (50)
Need for hospital admission	7/21 (33)	1/14 (7)	2/3 (67)	4/4 (100)
Need for intensive care unit admission	4/21 (19)	0	0	4/4 (100)
Decolonization performed	13/21 (62)	9/14 (64)	1/3 (33)	3/4 (75)
Recurrence after decolonization	2 ^##^ (10)	2/9 (22)	0	0

HIV: human immunodeficiency virus; USA: United States of America; TMP/SMX: trimethoprim/sulfamethoxazole. * One pediatric patient with septic shock due to cellulitis, osteomyelitis and compartment syndrome, and three adult patients with septic shock due to necrotizing pneumonia. ^°^ One patient was on antiretroviral therapy, suppressed and with a T_CD4+_ cell count >500/mm^3^; the other patient had no antiretroviral treatment and had a T_CD4+_ cell count of 154/mm^3^. ^#^ One patient had Hashimoto thyroiditis and the other ulcerative colitis; none of them had systemic immunosuppressive treatment. ^§^ An Italian patient, currently in Portugal for vacation. ^^^ Five patients came from Angola, two from Guinea Bissau and one from Mozambique. ^$^ Two patients, recently returned from vacations, one in Thailand and the other in India. ** Data only available for 11 patients (4 with severe disease, 2 with moderate disease, 5 with mild disease). No patient had absolute neutropenia. ^°°^ In the osteomyelitis patient, *S. aureus* was isolated both from pus and also from bone samples. ^##^ Both patients had probable secondary cases in close relatives who did not comply with decolonization recommendations.

**Table 2:** Antimicrobial resistances of PVL-SA isolates.

Antimicrobial	Total, n (%)	MSSA, n (%)	MRSA, n (%)
Oxacillin	5/21 (24)	0/21 (0)	5/21 (24)
TMP/SMX	6/21 (29)	5/21 (24)	1/21 (5)
Erythromycin	4/21 (19)	2/21 (10)	2/21 (10)
Tetracycline	3/21 (14)	2/21 (10)	1/21 (5)
Clindamycin	3/21 (14)	2/21 (10)	1/21 (5)
Rifampin	3/18 (17)	3/18 (17)	0/18 (0)
Gentamycin	1/21 (5)	0/21 (0)	1/21 (5)
Ciprofloxacin	1/20 (5)	0/20 (0)	1/20 (5)
Levofloxacin	1/21 (5)	0/21 (0)	1/21 (5)
Moxifloxacin	0/21 (0)	0/21 (0)	0/21 (0)
Vancomycin	0/21 (0)	0/21 (0)	0/21 (0)
Linezolid	0/19 (0)	0/19 (0)	0/19 (0)
Teicoplanin	0/21 (0)	0/21 (0)	0/21 (0)
Fusidic Acid	2/21 (10)	2/21 (10)	0/21 (0)
Mupirocin	0/6 (0)	0/6 (0)	0/6 (0)

MSSA: methicillin-susceptible *Staphylococcus aureus*; MRSA: methicillin-resistant *Staphylococcus aureus*; TMP/SMX: trimethoprim/sulfamethoxazole.

**Table 3:** Comparison between methicillin-sensitive *Staphylococcus aureus* and methicillin-resistant Staphylococcus aureus isolates.

	MSSA, n (%)	MRSA, n (%)	*p*-Value *
Recent travel history			1
Yes	9 (75)	3 (25)	
No	7 (78)	2 (22)	
Healthcare context			0.43
Yes	1 ^°^ (50)	1 ^§^ (50)	
No	15 (79)	4 (21)	
Previous antibiotic treatments			0.03
Yes	10 (83)	3 (33)	
No	2 (17)	6 (67)	

Notes: MSSA: methicillin-susceptible *Staphylococcus aureus*; MRSA: methicillin-resistant *Staphylococcus aureus*; * Fisher exact test. ^°^ Recent humanitarian work, as a doctor, in Mozambique. ^§^ Previous hospitalization in Guinea-Bissau.
